# Impact of Human Genetic Variation on C-Reactive Protein Concentrations and Acute Appendicitis

**DOI:** 10.3389/fimmu.2022.862742

**Published:** 2022-05-25

**Authors:** Isis Ricaño-Ponce, Toon Peeters, Vasiliki Matzaraki, Bert Houben, Ruth Achten, Peter Cools, Mihai G. Netea, Inge C. Gyssens, Vinod Kumar

**Affiliations:** ^1^ Department of Internal Medicine and Radboud Center for Infectious Diseases, Radboud University Medical Center, Nijmegen, Netherlands; ^2^ Department of Infectious Diseases & Immunity, Jessa Hospital, Hasselt, Belgium; ^3^ Faculty of Medicine and Life Sciences, Hasselt University, Hasselt, Belgium; ^4^ Department of General and Abdominal Surgery, Jessa Hospital, Hasselt, Belgium; ^5^ Department of Abdominal Surgery, GZA Hospital, Antwerpen, Belgium; ^6^ Human Genomics Laboratory, Craiova University of Medicine and Pharmacy, Craiova, Romania; ^7^ Department of Genetics, University of Groningen, University Medical Center Groningen, Groningen, Netherlands; ^8^ Nitte (Deemed to be University), Nitte University Centre for Science Education and Research (NUCSER), Deralakatte, Mangalore, India

**Keywords:** C-reactive protein (CRP), appendicitis, functional genetics, biomarker, quantitative trait

## Abstract

**Background:**

Acute appendicitis is one of the most common abdominal emergencies worldwide. Both environmental and genetic factors contribute to the disease. C-reactive protein (CRP) is an important biomarker in the diagnosis of acute appendicitis. CRP concentrations are significantly affected by genetic variation. However, whether such genetic variation is causally related to appendicitis risk remains unclear. In this study, the causal relationship between single-nucleotide polymorphisms (SNPs) associated with circulating CRP concentrations and the risk and severity of acute appendicitis was investigated.

**Methods:**

CRP concentrations in serum of appendicitis patients (n = 325) were measured. Appendicitis was categorized as complicated/uncomplicated and gangrenous/non-gangrenous. Imputed SNP data (n = 287) were generated. A genome-wide association study (GWAS) on CRP concentrations and appendicitis severity was performed. Intersection and colocalization of the GWAS results were performed with appendicitis and CRP-associated loci from the Pan-UKBB cohort. A functional-genomics approach to prioritize genes was employed.

**Results:**

Thirteen percent of significant CRP quantitative trait loci (QTLs) that were previously identified in a large cohort of healthy individuals were replicated in our small patient cohort. Significant enrichment of CRP-QTLs in association with appendicitis was observed. Among these shared loci, the two top loci at chromosomes 1q41 and 8p23.1 were characterized. The top SNP at chromosome 1q41 is located within the promoter of H2.0 Like Homeobox (*HLX*) gene, which is involved in blood cell differentiation, and liver and gut organogeneses. The expression of *HLX* is increased in the appendix of appendicitis patients compared to controls. The locus at 8p23.1 contains multiple genes, including cathepsin B (*CTSB*), which is overexpressed in appendix tissue from appendicitis patients. The risk allele of the top SNP in this locus also increases *CTSB* expression in the sigmoid colon of healthy individuals. *CTSB* is involved in collagen degradation, MHC class II antigen presentation, and neutrophil degranulation.

**Conclusions:**

The results of this study prioritize *HLX* and *CTSB* as potential causal genes for appendicitis and suggest a shared genetic mechanism between appendicitis and CRP concentrations.

## Introduction

Acute appendicitis is one of the most common surgical emergencies worldwide (1). The lifetime risk is estimated at between 6% and 17% (2, 3). Acute appendicitis involves inflammation of the appendix and can be associated with serious complications such as localized abscess, peritonitis, and perforation. Depending on the severity of the disease and presence of complications, appendicitis is often referred to as complicated or uncomplicated. Based on histological examination for the presence of necrosis, the disease can also be classified as gangrenous or non-gangrenous.

The etiology of the disease is likely multifactorial. Positive family history is often observed in patients, indicating that appendicitis can be a consequence of both genetic and environmental risk factors. Segregation analyses have shown that appendicitis is inherited by a polygenic model in which several genetic loci, in addition to environmental triggers, contribute to the disease phenotype (4). Twin studies and population-based studies also show familial transmission of acute appendicitis through genetic as well as environmental effects (5–7).

The role of genetic factors in the development of acute appendicitis has been confirmed in a number of candidate gene-based as well as genome-wide association studies (GWASs). Although inconsistent associations exist between the severity of appendicitis and single-nucleotide polymorphisms (SNPs) at *NOD2*/*CARD15* (8) and IL-6 genes (9, 10), a significant association at SNP rs2129979 near *PITX2* gene has been observed in adults (11). This finding was confirmed in another study, and a number of additional susceptibility genes were identified (12). Among other genes, *PITX2* was also differentially expressed in different categories of appendiceal inflammation, suggesting a potential genetic link between inflammation regulation and appendicitis phenotypes.

The etiology of appendicitis is not yet well understood; however, several observations point to the pathogenic pathways related to inflammation. C-reactive protein (CRP) is one of the most abundant systemic markers of inflammation and one of the most important biomarkers in the diagnosis of acute appendicitis. In addition, serum CRP concentrations appear to be associated with disease severity (13–16). With the use of QTL mapping strategies in large population-based cohorts, it has been shown that the CRP concentrations are significantly affected by host genetic variation (17, 18). However, whether such CRP-QTLs are causally related to appendicitis risk remains unclear. Therefore, in this study, the causal relationship between SNPs associated with circulating CRP concentrations and the risk and severity of acute appendicitis was investigated.

## Materials and Methods

### Study Population

All patients included in this study gave written informed consent before participation; in the case of children, informed consent was obtained from parents or guardians. The study was approved by the Medical Ethics Committee of Jessa Hospital, Hasselt, Belgium. The study was registered at Clinicaltrials.gov under identifier NCT02391675. The study population consisted of 374 appendicitis patients (**Table 1**). A total of 325 patients were prospectively recruited in the HAPPIEST population at Jessa Hospital, Hasselt, Belgium, between June 2012 and October 2016. An additional 49 patients were recruited at Sint-Vincentius Hospital, Antwerp, Belgium, between September 2015 and March 2017. Patients between the ages of 5 and 85 were considered possible candidates. All included patients had an appendectomy in the first 5 days after diagnosis. Exclusion criteria were patients with an appendectomy more than 5 days after onset of symptoms, pregnant, and immunocompromised patients.

**Table 1:** Demographics of patients in the HAPPIEST cohort.

Demographics	HAPPIEST cohort (n = 374)	Uncomplicated (n = 260)	Complicated (n = 114)	Non-gangrenous (n = 258)	Gangrenous (n = 116)
	n (%)	n (%)	n (%)	n (%)	n (%)
Gender					
Male	198 (52.9)	129 (49.6)	69 (60.5)	131 (50.8)	67 (57.8)
Female	176 (47.1)	131 (50.4)	45 (39.5)	127 (49.2)	49 (42.2)
Age, mean ± SD (range)	32.8 ± 17.8 (5–81)	30.8 ± 16.9 (5–81)	37.2 ± 19.2 (5–79)	30.4 ± 16.1 (5–75)	38.0 ± 20.4 (5–81)

Acute appendicitis was diagnosed based on clinical symptoms, laboratory results, and ultrasound and/or CT scan. Clinical data and medical history of patients were recorded. Patients received standard-of-care treatment. CRP concentrations were measured as routine at the time of admission at Jessa Hospital. Upon surgery, the appendix was sectioned, and appendicitis was classified by the surgeon according to ICD-9 codes. Samples from a 1-cm part of the tip, the middle, and the base were sent to the pathology department to confirm the diagnosis and assign histological severity (gangrenous vs. non-gangrenous). Acute appendicitis with general peritonitis (540.0) or peritoneal abscess (540.1) was considered complicated; patients with no data on abscess or peritonitis (540.9) were considered uncomplicated. In the HAPPIEST population, microbiological and immunological factors contributing to the risk and severity of acute appendicitis were studied previously (16, 19).

### Sample Collection

During surgery, EDTA blood was collected (3 × 10 ml in the case of adults and 2 × 10 ml in the case of children). Samples were aliquoted and frozen at −80°C at the University Biobank Limburg (UBiLim). DNA isolation from whole blood was performed using the DNeasy Blood and Tissue kit (Qiagen, Venlo, the Netherlands) according to the manufacturer’s instructions. DNA samples were stored at −20°C until further analysis.

### Genotyping, Quality Control, and Imputation

The study design is presented in **Supplementary Figure 1** (**Additional File 1**). DNA samples from 331 individuals who passed quality control (QC) were hybridized on the Infinium CoreExome v3 SNP chip from Illumina. Genotype calling was performed using optiCall 0.7.0 using default settings (20). Non-polymorphic markers and markers within sexual chromosomes were excluded for further analysis. Chromosome coordinates are given in human Build 37 (hg19). QC checks and filters were performed using PLINK version 1.09 (21). Samples with a call rate <99% and SNPs with a call rate <99%, Hardy–Weinberg equilibrium exact test ≤0.001, and minor allele frequency (MAF) ≤0.001 were discarded. Three individuals were removed because of an excess of homozygosity rate. Two individuals were removed due to hidden relatedness calculated by identity-by-descent estimates. Thirty-nine population outliers were detected by multidimensional scaling (MDS) plots using R Studio and removed for further analysis. After QC, the dataset comprised 287 samples and 272,017 SNPs. Strands of variants were aligned and identified against the 1000 Genomes reference panel using Genotype Harmonizer (22). Genotype imputation was performed on the Michigan Imputation Server using the human reference consortium as a reference panel, and variants with an R^2^ ≤ 0.3 and MAF ≤ 0.10 were excluded for imputation QC (23).

### Genome-Wide Association Studies

GWAS on complicated versus uncomplicated appendicitis and gangrenous vs. non-gangrenous was performed using SNPtest version 2.5.2 (24). A frequentist additive model was run including age and sex as covariates. To control for genotype uncertainty, the parameter score was selected to include a missing data likelihood score test.

### Prioritization of Shared Loci Between C-Reactive Protein and Appendicitis

To test whether CRP and appendicitis had shared genetic loci, the summary statistics from the Pan-ancestry genetic analysis of the UK Biobank (Pan-UKBB) were used (25). The files were extracted according to their phenotype manifest. The Pan-UKBB ID in the manifest for the CRP-QTLs in the general population is “biomarkers-30710” and for acute appendicitis “phecode-540.1”. All analyses were focused on the results from individuals with European ancestry. The CRP-QTL study was a population-based study (n = 400,094), where the appendicitis cohort comprised 3,490 acute appendicitis cases and 416,195 controls. SNP IDs were annotated based on the variant manifest file provided by Pan-UKBB.

Expression QTLs were obtained from the GTEx portal (26) on February 13, 2020. Microarray gene expression from the appendix of 9 appendicitis cases and 4 individuals who were undergoing intra-abdominal surgery for a non-inflammatory condition has been generated previously (27), and it is publicly available at Gene Expression Omnibus with ID “GSE9579.” Expression values were obtained using GEO2R on the same website; the probe selected for HLX was 214,438_AT, and for CTSB, it was 200838_AT. The expression values were compared using a t-test. Chromatin states were annotated using data from sigmoid colon and colon smooth muscle reported by the Roadmap Epigenomics Mapping Consortium (28). These data were obtained and visualized on the WashU epigenome browser (29).

### Quantitative Trait Locus Mapping of C-Reactive Protein Concentrations in Appendicitis Patients

CRP concentrations and genotypes for 285 individuals were available. Normalization of CRP concentrations was done by inverse rank quantile normalization. The correlation between normalized CRP concentrations and genotype was assessed with a linear regression model including age and sex as covariables using the eQTLMatrix package in R. Significance was considered as p < 0.05.

### Statistical Analysis

CRP concentrations in different age groups were compared by dividing the cohort into four different age range groups. The difference in CRP concentrations was assessed using the non-parametrical Kruskal–Wallis sum rank test, followed by a Dunn’s test to compare between groups. To assess the shared genetic variants between appendicitis and CRP concentrations, the genome-wide significant CRP-QTLs were extracted from the Pan-UKBB and intersected with the results from the appendicitis GWAS performed in the same cohort. The enrichment was calculated based on the quantification of the degree of inflation between the observed and expected p-values from a uniform distribution in the QQ plot. Statistical analysis and plots were generated using R Studio. Genome-wide significance was defined as p < 5 × 10^−8^, suggestive association as p < 1 × 10^−5^, and significance as p < 0.05. All top SNPs in the loci were functionally annotated using SNP Nexus (https://snp-nexus.org/), including the predicted effect of non-synonymous coding SNPs on protein function by SIFT and PolyPhen. Gene Ontology (GO) terms and PathCard information for the candidate genes were extracted from GeneCards^®^: The Human Gene Database (https://www.genecards.org).

## Results

### C-Reactive Protein Quantitative Trait Loci in Appendicitis Patients

From the 374 appendicitis patients included in the study, the mean age was 32.8 (range 5–81), and 198 patients (52.9%) were male. **Table 1** shows the age and sex of patients with complicated appendicitis, non-complicated appendicitis, and gangrenous and non-gangrenous appendicitis. To assess the difference in CRP concentration at different ages, we stratified the cohort into four different groups, as shown in **Supplementary Figure 2** (**Additional File 2**). CRP concentration was significantly different between groups (p = 3.24 × 10^−6^) and seems to be increasing with age. All groups showed significant differences, except when the group below 20 years old was compared with the group between 20 and 40 years old (p = 0.14) and patients between 40 and 60 years old were compared with patients between 60 and 80 years old.

To test whether the previously reported genetic causes of CRP concentrations (CRP-QTLs) in healthy individuals can be replicated in appendicitis patients, QTL mapping in the cohort was performed. The analysis included 256 individuals in whom we had imputed SNP data and CRP measurements. After 4,416,143 SNPs were tested, 8 loci with suggestive association were identified. Our results were compared with significant CRP-QTLs from the European population in the Pan-UKBB cohort (n = 400,094). Of 65,457 SNPs significantly associated with CRP concentrations in the Pan-UKBB cohort, 36,042 SNPs were present in our analysis, and 4,754 (13.2%) were replicated with a p < 0.05 (**Supplementary Table 1**).

### Shared Loci Between C-Reactive Protein and Appendicitis in the Pan-UKBB Cohort

**Figure 1:**
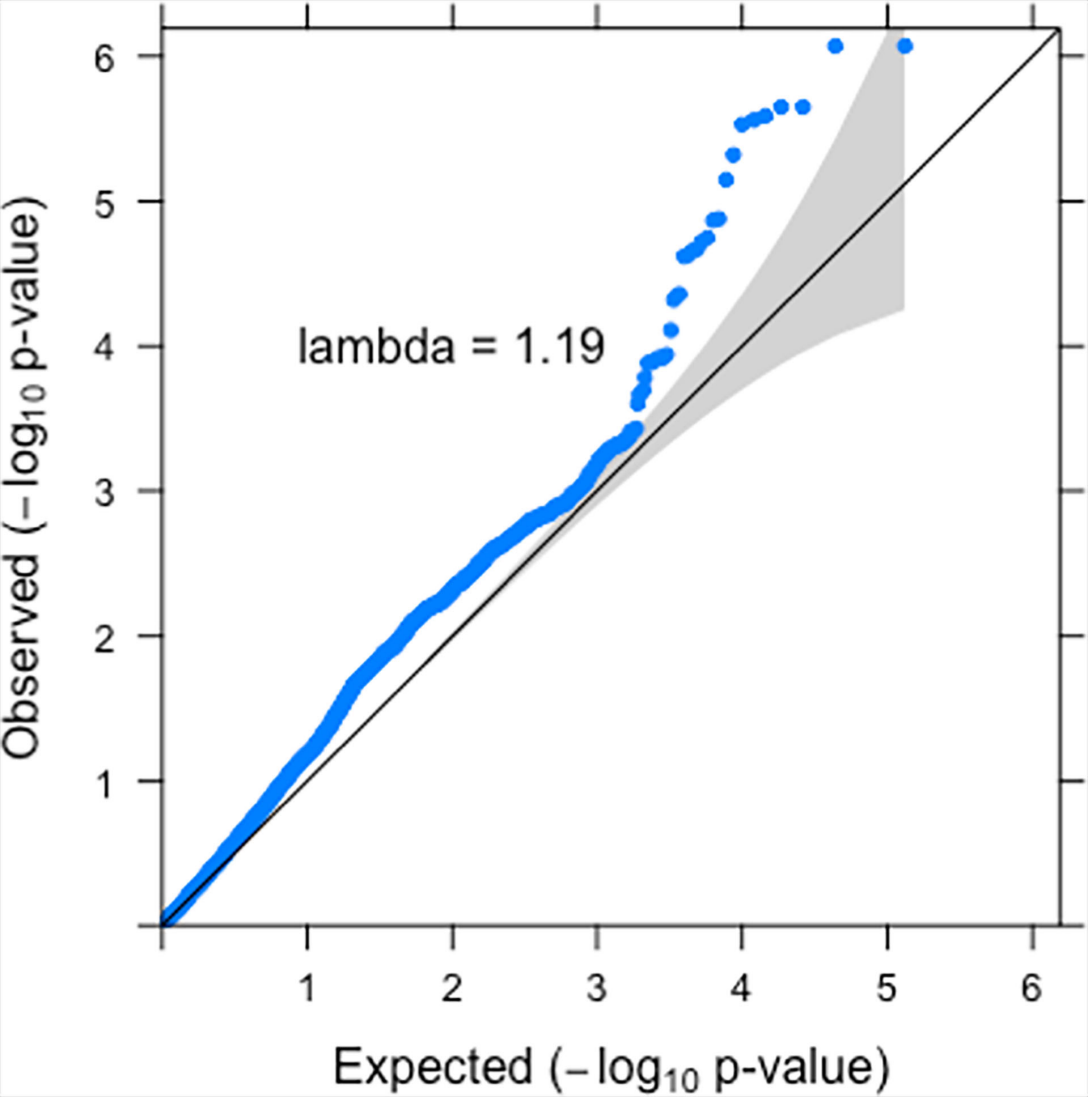
Enrichment of CRP-QTLs in appendicitis GWAS. CRP, C-reactive protein; QTLs, quantitative trait loci; GWAS, genome-wide association study.

To test whether CRP concentrations are genetically related to acute appendicitis, we looked for enrichment of CRP-associated SNPs in appendicitis GWAS. For this analysis, 65,457 genome-wide significant CRP-QTLs from the Pan-UKBB cohort were intersected with the summary statistics from the appendicitis GWAs performed in the same cohort. The analysis revealed significant enrichment of CRP-associated SNPs in appendicitis GWAS (inflation factor of 1.19), as shown in **Figure 1**.

From the 65,457 SNPs, two loci on chromosomes 1q41 and 8p23.1 reached suggestive association (p < 1 × 10^−5^) in appendicitis GWAS. To further characterize these loci, functional annotation of the SNPs was performed using literature as well as genomic information from the publicly available databases.

### Prioritization of *HLX* as a Potential Causal Gene on Chromosome 1q41

**Figure 2:**
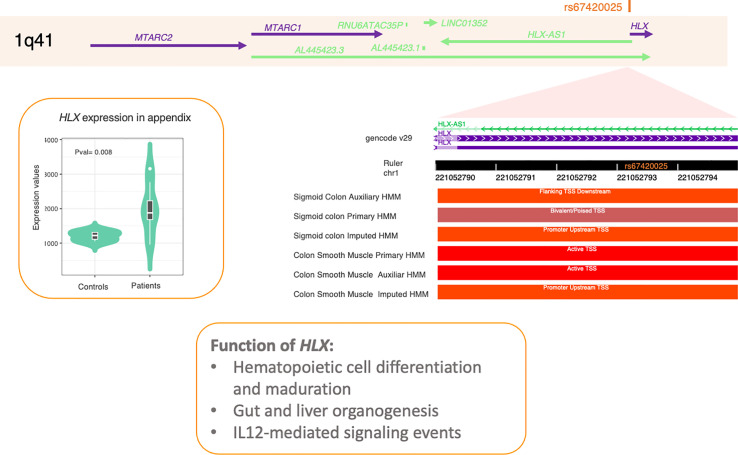
Locus on chromosome 1q41. SNP rs67420025 shows a suggestive association to appendicitis in the Pan-UKBB cohort, and it is located in the promoter region of *HLX* and overlaps a transcription start site (TSS) in colon smooth muscle. The right panel shows a significant increase in *HLX* expression in the appendix of 9 appendicitis patients and 4 controls. The box at the bottom shows the function of *HLX* as reported in the literature and PathCards. SNP, single-nucleotide polymorphism.

The strongest association in chromosome 1q41 was SNP rs67420025 (p = 7.15 × 10^−6^). This SNP is located in the promoter region of the H2.0 Like Homeobox (*HLX*) gene and overlaps a transcription start site characterized in sigmoid colon and colon smooth muscle, as depicted in **Figure 2**. rs67420025 is in high linkage disequilibrium (LD) (r^2^ = 0.928) with rs2738755, a missense variant in *HLX* that leads to amino acid proline-to-arginine change or proline-to-leucine change, depending on the transcript (NP_068777.1:p.Pro356Arg and NP_068777.1:p.Pro356Leu). While the change from proline to arginine is predicted to be benign by PolyPhen and tolerated by SIFT, the change to leucine is predicted to be deleterious by SIFT.

Moreover, gene expression of *HLX* is significantly higher in the appendix of appendicitis patients compared to that of individuals who were undergoing intra-abdominal surgery for a non-inflammatory condition (p = 0.008, **Figure 2**). According to the GO biological process, *HLX* is involved in cell differentiation, embryonic digestive tract morphogenesis, enteric nervous system development, liver development, multicellular organism development, negative regulation of T-helper 2 cell differentiation, positive regulation of cell population proliferation, positive regulation of organ growth, positive regulation of T-helper 1 cell differentiation, and skeletal muscle tissue development. A study using a mouse model with deficient expression of *HLX* revealed that *HLX* is also involved in liver and gut morphogenesis (30). Additionally, PathCards reports *HLX* as part of the IL-12-mediated signaling events SuperPath.

In an independent acute appendicitis GWAS performed on the FinnGen cohort (31), the *HLX* locus on chromosome 1q41 has reached genome-wide significance (top SNP = rs3738182, p = 7.1 × 10^−10^). Although the top SNP rs67420025 was not tested in the FinnGen cohort, its proxy rs2738755 reaches genome-wide significance in the FinnGen analysis (p = 3.6 × 10^−8^). These observations indicate the highly likely causal role of *HLX* in conferring susceptibility to appendicitis.

### Prioritization of *CTSB* as a Potential Causal Gene on Chromosome 8p23.1

**Figure 3:**
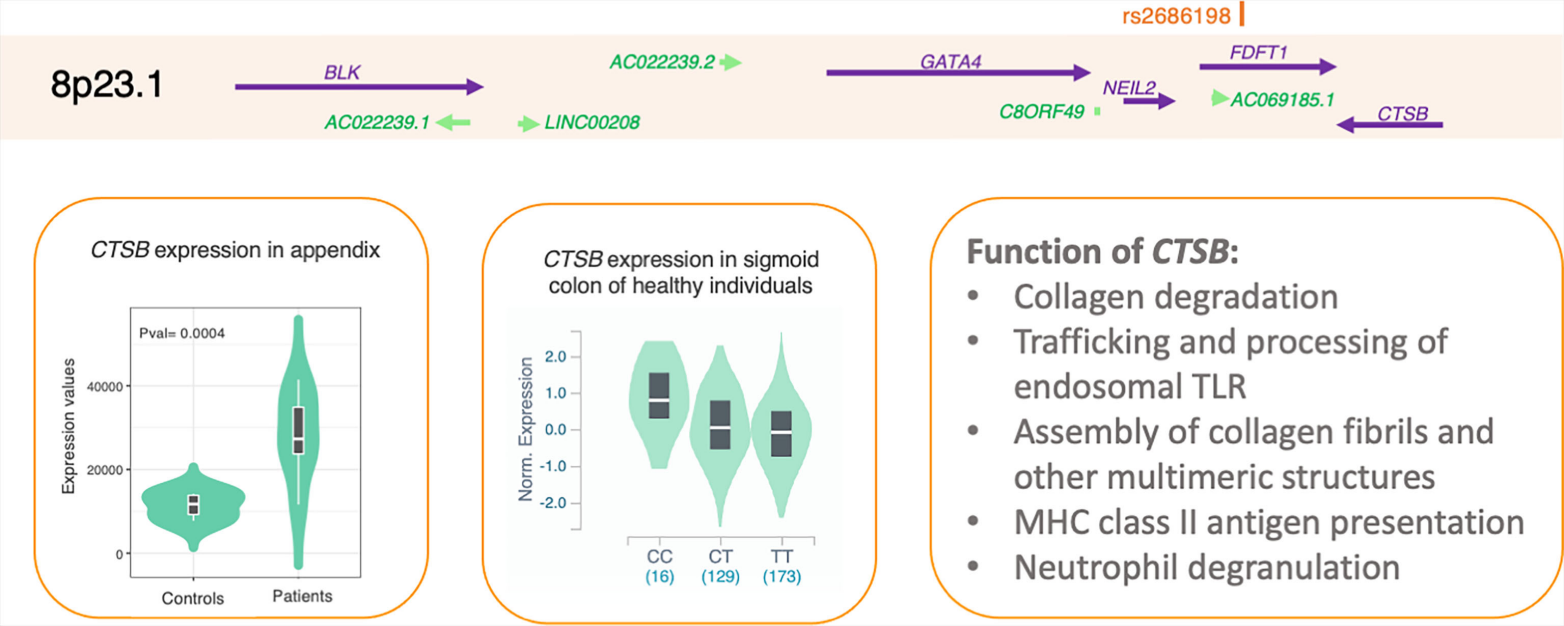
Locus on chromosome 8p32.1. The strongest association in this locus corresponds to SNP rs2686198 with a p-value of 8.48 × 10−7 in the Pan- UKBB cohort. There are multiple genes in the locus, but only cathepsin B (CTSB) is significantly overexpressed in the appendix of patients compared to individuals who were undergoing intra-abdominal surgery for a non-inflammatory condition, as depicted in the left panel. The risk allele of rs2686198*C is associated with higher expression of CTSB in sigmoid colon in healthy individuals. The left panel shows the function of CTSB according to Reactome. SNP, single-nucleotide polymorphism.

The top SNP on chromosome 8p32.1 locus is rs2686198 (p = 8.48 × 10^−7^). This SNP is located in an intron of the farnesyl-diphosphate farnesyltransferase 1 (*FDFT1*) gene. The locus contains multiple genes including cathepsin B (*CTSB*) gene (**Figure 3**). The expression of CTSB was significantly higher in the appendix of appendicitis patients compared to individuals who were undergoing intra-abdominal surgery for a non-inflammatory condition (p = 0.0004). None of the other genes within the locus showed a significant difference in this comparison. Additionally, the risk allele (C) of rs2686198 significantly increases the expression of *CTSB* in the sigmoid colon of individuals from the GTEx project (p = 2.7 × 10^−11^) and the CTSB concentrations in plasma (p = 1.9 × 10^−28^) (32).

According to Reactome, *CTSB* is involved in collagen degradation, trafficking and processing of endosomal TLR, assembly of collagen fibrils and other multimeric structures, MHC class II antigen presentation, and neutrophil degranulation. The inflammasome pathway, which includes CTSB, was significantly upregulated in plasma from appendicitis patients compared to patients with sexually transmitted infection-induced endometritis (adjusted p = 0.0017) (33). These observations point to the important role of *CTSB* in regulating inflammation and in turn affecting susceptibility to appendicitis.

### Shared Loci on Appendicitis and Disease Severity in the Belgian Cohort

In order to establish whether genetic variants associated with appendicitis also predispose to disease severity, a GWAS was performed on our Belgian cohort, comparing complicated patients with uncomplicated appendicitis patients and gangrenous with non-gangrenous appendicitis. A total of 331 individuals in whom DNA was available were genotyped. After QC filtering per sample and per SNPs, 287 individuals were imputed. After imputation QC, 430,122 SNPs and 285 individuals were available for the GWAS analysis.

For the GWAS on appendicitis complications, the individuals were split into complicated appendicitis cases (n = 82) and uncomplicated cases (n = 203). No loci reaching genome-wide significance were identified, although 11 loci showed a suggestive association (p < 1 × 10^−5^, **Supplementary Table 2**).

To assess the genetic influence on the development of gangrenous appendicitis, a GWAS was performed including 91 patients with gangrenous appendicitis and 194 patients with non-gangrenous appendicitis. Although ten loci with suggestive associations were found, none of them reached genome-wide significance (**Supplementary Table 3**).

The 585 SNPs showing suggestive associations with appendicitis identified in the PAN-UKBB cohort were intersected with our results. Out of 277 SNPs tested in this cohort, only 46 SNPs showed a nominal association. One SNP (rs61571009, p in the Belgian cohort = 0.037, p in the PAN-UKBB = 1.13 × 10^−7^) was associated with appendicitis complications, while 45 comprising two loci were associated with gangrenous appendicitis (**Supplementary Table 4**).

## Discussion

Given the pleiotropic nature of many molecular phenotypes, one of the important observations of this study is the identification of shared genetic loci between CRP concentrations and appendicitis. The fact that 13.2% of significant CRP-QTLs that were identified previously in a large cohort of healthy individuals could be replicated in a small patient cohort and the significant enrichment of CRP-QTLs in appendicitis GWAS suggests the causal role of CRP in the disease and bridge a knowledge gap regarding SNP–disease associations in appendicitis.

Among the two prioritized causal genes, the strongest association was found in *HLX* gene. A recent study in the FinnGen cohort (with a four times higher sample size than the Pan-UKBB; 12,476 acute appendicitis patients and 163,396 controls) has also identified this locus to be associated with appendicitis, confirming its causal role in the disease. In this study *HLX* was prioritized as a potential causal gene at this locus, using multiple lines of evidence. The top SNP overlaps a transcription start site of *HLX*, and the gene is overexpressed in the appendix of appendicitis patients. Additionally, one of the proxies is a missense variant in the same gene. Although we found that one of the proxies of the top SNP had multiple expression QTLs (eQTLs) reported, including HLX in the brain, so far there are no eQTLs determined in the appendix. As eQTLs are highly tissue- and context-specific, such associations in the appendix should be considered in future studies.

The second prioritized gene, *CTSB*, encodes cathepsin B protein. We found that the top SNP at this locus increases the expression of *CTSB* in the sigmoid colon in healthy individuals, showing a regulatory effect of this SNP. Moreover, this SNP also increases the concentrations of CTSB protein in the plasma of healthy individuals. Interestingly, *CTSB* is predicted to interact with the previously established appendicitis gene *PITX2* by the Integrated Interactions Database version 2021-05 (11, 12, 34). Therefore, it is tempting to speculate that *CTSB* and *PITX2* may affect similar molecular pathways to cause appendicitis.

There are also a number of limitations to this study. Firstly, the patient sample size is relatively small, which limited the ability to draw strong conclusions from GWAS of appendicitis complications. Although the shared genetics between appendicitis susceptibility and disease severity was assessed, only 46 SNPs reached a nominal significance in our cohort. As it is shown in the context of immune diseases—for example, in inflammatory bowel disease (IBD) and celiac disease, SNPs conferring risk to disease susceptibility are different from the ones predisposing to disease severity (35, 36)—it could be interesting for future research to test this hypothesis in a larger appendicitis cohort, as it might reflect the difference in the tested sample sizes. Secondly, the gene expression data from the appendix of appendicitis patients and controls were generated using micro-array technology that did not include the majority of non-coding genes. Therefore, their role in appendicitis cannot be excluded. Finally, our cohort comprises patients within an age range from 5 to 81 years, although correcting for age in our analysis addresses this issue; by pooling all age groups together, we might dilute the power to identify age-specific genetic effects. This could potentially affect the comparison that we performed with the Pan-UKBB cohort, as the age range from this cohort is 40 to 69. The age restriction in this cohort was due to the primary aim of the study: to improve the prevention, diagnosis, and treatment of serious illnesses that typically onset later in life, including diabetes, cancer, arthritis, heart disease, stroke, and dementia. Similarly, previous GWASs in appendicitis susceptibility have been performed only using adult patients. Thus, further studies need to be performed in different age ranges to assess if the extent of shared genetics of appendicitis and CRP varies with different age groups.

## Conclusions

Our results prioritize *HLX* and *CTSB* as potential causal genes for appendicitis and suggest a shared genetic mechanism between appendicitis and CRP concentrations.

## Data Availability Statement

The datasets presented in this article are not readily available because the informed consent did not include permission for genotype sharing. Requests to access the datasets should be directed to Vinod Kumar at V.Kumar@radboudumc.nl.

## Ethics Statement

The studies involving human participants were reviewed and approved by the Medical Ethics Committee of Jessa Hospital, Hasselt, Belgium. The patients/participants provided their written informed consent to participate in this study. In the case of children informed consent was obtained from parents or guardians.

## Author Contributions

ICG and VK designed and supervised the study. BH, PC, TP recruited the patients, BH and PC collected the samples and coded the clinical severity and RA performed the histological analysis. IR-P, TP and VM performed the analyses. IR-P and TP wrote the initial manuscript, MGN, ICG, BH, PC, RA, VM and VK reviewed and edited the manuscript. All authors read and approved the final manuscript.

## Funding

This study is part of the Limburg Clinical Research Program (LCRP) UHasselt-ZOL-Jessa, supported by the foundation Limburg Sterk Merk, province of Limburg, Flemish government, Hasselt University, Ziekenhuis Oost-Limburg, and Jessa Hospital. Part of the work was funded by a research grant (2017) from the European Society of Clinical Microbiology and Infectious Diseases, a Radboudumc Hypatia Grant (2018), and a grant from the research program ZonMw COVID-19 call by the Netherlands Organisation for Health Research and Development (ZonMw) to VK.

## Conflict of Interest

The authors declare that the research was conducted in the absence of any commercial or financial relationships that could be construed as a potential conflict of interest.

## Publisher’s Note

All claims expressed in this article are solely those of the authors and do not necessarily represent those of their affiliated organizations, or those of the publisher, the editors and the reviewers. Any product that may be evaluated in this article, or claim that may be made by its manufacturer, is not guaranteed or endorsed by the publisher.
