# Supplementary figures and images for: Impact of Human Genetic Variation on C-Reactive Protein Concentrations and Acute Appendicitis
Source: Front Immunol. 2022 May 25;13:862742. doi: 10.3389/fimmu.2022.862742 (PMC9174512; doi:10.3389/fimmu.2022.862742)

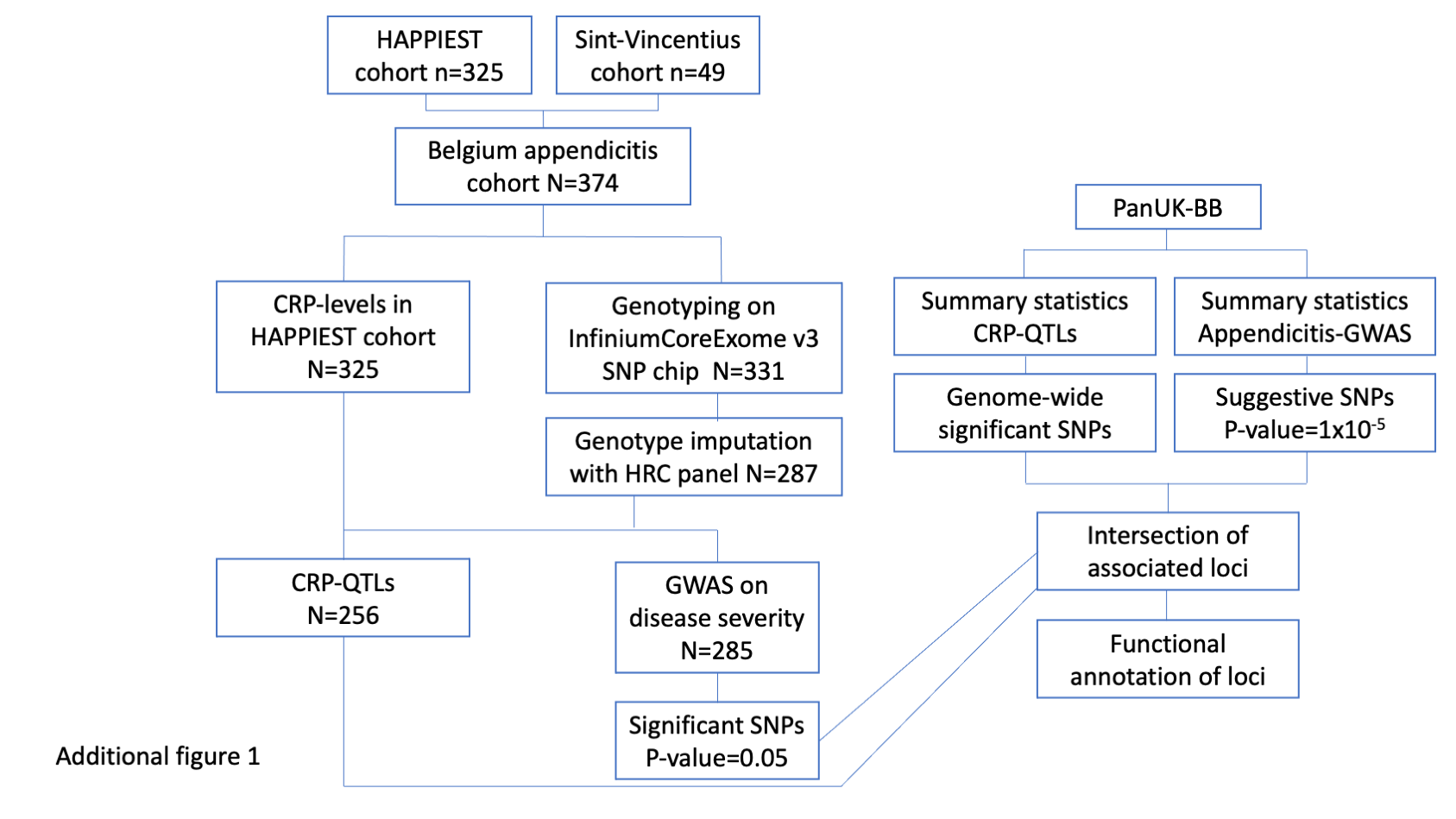

Supplement: Supplementary file 2 [file Image_1.tiff]

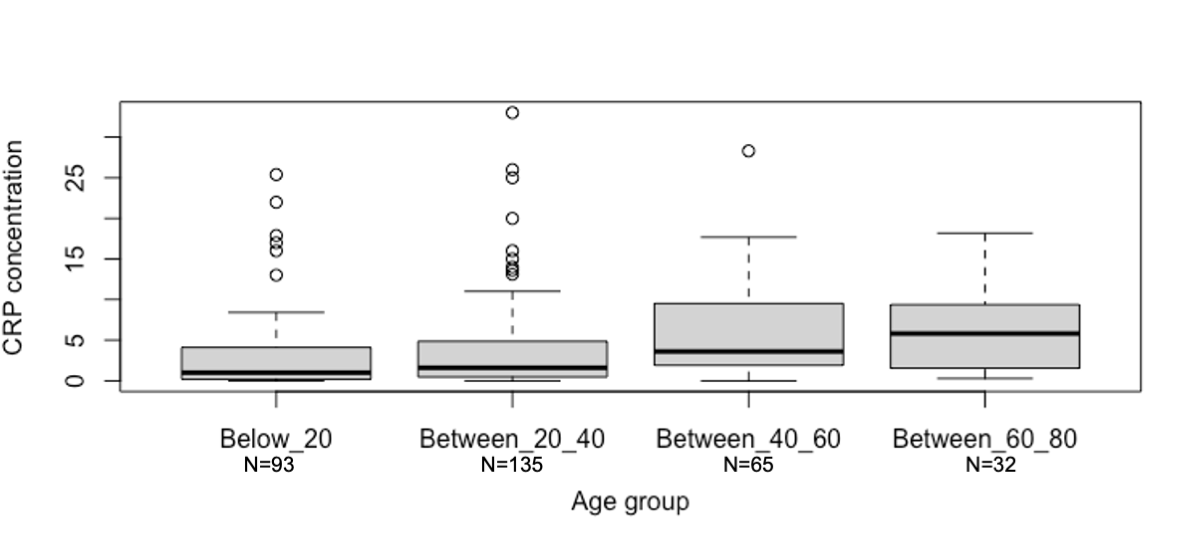

Supplement: Supplementary file 3 [file Image_2.tiff]
